# Enamel evaluation by scanning electron microscopy after debonding brackets and removal of adhesive remnants

**DOI:** 10.4317/jced.54553

**Published:** 2018-03-01

**Authors:** Dikson Claudino, Milton-Carlos Kuga, Lauriê Belizário, Jefferson-Ricardo Pereira

**Affiliations:** 1 PhD student, Postgraduate Program in Health Sciences, University of Southern Santa Catarina, Tubarão, SC, Brazil; 2 Associate Professor, Restorative Dentistry Department, Araraquara Dental School, São Paulo State University, Araraquara, SP, Brazil; 3 PhD student, Restorative Dentistry Department, Araraquara Dental School, São Paulo State University, Araraquara, SP, Brazil; 4 Associate Professor, Postgraduate Program in Health Sciences, University of Southern Santa Catarina, Tubarão, SC, Brazil

## Abstract

**Background:**

The bonding of accessories in the dental crown during the orthodontic treatment creates microporosities, thus promoting micromechanical retention of the adhesive to the enamel structure. After debonding brackets, at the end of the active orthodontic treatment, a certain amount of adhesive remnants must be mechanically removed from the enamel. The objective of this study was to compare, by means of scanning electron microscopy, three different methods to remove the adhesive remnants after orthodontic bracket removal.

**Material and Methods:**

An experimental analytical study was conducted on human premolar specimens, extracted within a year or less. The preparation of the enamel was carried out with the application of 35% phosphoric acid and Transbond XT Light Cure Adhesive Primer® adhesive. Edgwise Standart prescription brackets, slot .022 “(Morelli Orthodontia) were glued to the enamel using Transbond XT® bonding resin. The brackets were placed on the center of the vestibular face of the clinical crown, and a 300-gram pressure was exerted against the surface of the enamel, measured with an orthodontic dynamometer. The brackets were debonded with adhesive removing pliers, and the samples were divided into groups, according to the protocol used for adhesive remnant removal: high-speed multi-laminated drill bit, low-speed multi-laminated drill bit, and low-speed glass fiber. After removal of the adhesive remnants, the samples went through scanning electron microscopy, obtaining electro micrographs with a magnification range of 150 X, 500 X, and 2,000 X.

**Results:**

The tested method showed that the best effectiveness for the removal of the adhesive remnants after bracket debonding was the use of a tungsten carbide multi-laminated high speed, followed by the use of a tungsten carbide multi-laminated, low-rotation drill. The use of fiberglass drill alone has proved to be inefficient for clinical use, given the large amounts of adhesive remnants it leaves on the enamel.

**Conclusions:**

All methods evaluated in this study proved to be inefficient for total removal of adhesive remnants from the enamel.

** Key words:**Dental enamel, microscopy, orthodontics.

## Introduction

Orthodontic treatment with fixed appliances has diffused widely in contemporary society. This type of treatment is based on the bonding of accessories in the dental crown, by means of acid etching of the enamel surface, which creates microporosities, thus promoting micromechanical retention of the adhesive to the enamel structure (1).

After detachment of the brackets, at the end of the active orthodontic treatment, a certain amount of adhesive remnants must be mechanically removed from the enamel, as they favor bacterial plaque retention and create color change over time (2).

Many studies have evaluated surface smoothness and roughness characteristics of dental enamel after bracket detachment and cleaning and polishing procedures (3-8). However, studies have not analyzed the presence and thickness of the adhesive remnant incorporated into the enamel structure after applying these methods (9).

As the penetration of the adhesive into the dental enamel occurs at different depths, varying amounts of the adhesive may remain embedded in the enamel structure after the mechanical removal of the adhesive remnants (6).

The objective of this study was to compare three different methods for removing the adhesive remnants from the enamel structure after orthodontic bracket debonding by using scanning electron microscopy. The working hypothesis stated there would be a statistically significant difference between the groups evaluated.

## Material and Methods

An experimental analytical study was carried out in a laboratory using human premolars extracted within a year or less, divided into three groups composed of three teeth each, obtained from the tooth bank of the dental school of the University of Southern Santa Catarina. Samples were excluded from the study if they had dental caries, restorations, fractures or cracks in the vestibular surface, and those with previous orthodontic treatment using bracket attachments, enamel shape changes, and blemishes caused by fluorosis, hypoplasia, or tetracycline.

Initially, the samples were cleaned by removing the periodontal remnants from the root surface using Gracey curettes and polishing the labial surface of the crown using a low-rotating rubber bowl and pumice paste. 

The dental enamel was prepared by drying the vestibular surface of the crown, using a triple syringe air jet for 10 seconds and subsequent application of 35% phosphoric acid (Dentsply, Rio de Janeiro, RJ, Brasil) for 15 seconds. The phosphoric acid was removed using a three-necked syringe water jet for 10 seconds, and air-jet drying for another 10 seconds. Then, the Transbond XT Light Cure Adhesive Primer® (3M/Unitek, Monrovia, CA, EUA) adhesive was applied by rubbing the surface with a microbrush making circular movements for 5 seconds. After the application, the adhesive was photopolymerized using an Optilight LD Max® (Gnatus, Ribeirão Preto, SP, Brasil) device with a measured power of mW/cm2, for 30 seconds, the light-curing tip being 1 mm apart from the dental surface.

**Figure 1:**
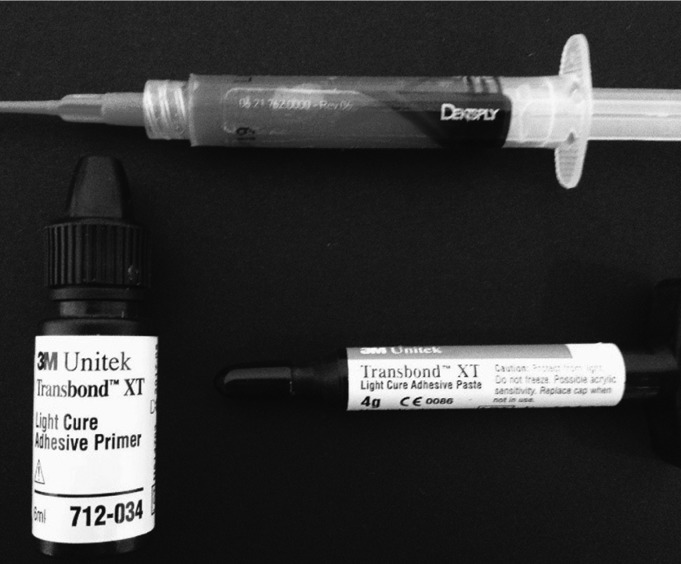
Adhesive system for bonding brackets.

After preparing the enamel samples, premolar brackets were bonded as prescribed by Edgwise Standart, slot .022” (Morelli Ortodontia, Sorocaba, SP, Brasil) using Transbond XT® (3M/Unitek) resin (Fig. 1). The amount of resin was measured (2 mm) using an endodontic ruler. The bracket was placed in the center of the vestibular clinical crown of the samples using an orthodontic pincer for brackets, and pressure of 300 grams was exerted against the surface of the enamel, measured with an orthodontic dynamometer (Correx Co, Waltham, MA, EUA). The resin excess in the edges of the bracket base was removed using an exploratory probe.

 After finishing bracket bonding, the adhesive bonding was photopolymerized for 40 seconds, positioning the light curing tip 1 mm apart from the tooth, for 20 seconds on the mesial surface, and another 20 seconds on the distal face of the brackets.

After bracket bonding, grooves were made on the enamel surface, parallel to the edges of the base of the brackets, using a 0.10 mm thick double-sided diamond disk (KG Sorensen) for subsequent cutting orientation and microscopy. The samples were then stored in serum at room temperature.

Twenty-four hours after gluing, the brackets were detached using 364R (Quinelato, Rio Claro, SP, Brasil) bracket remover pliers. The active plier tips were positioned in the vertical (occlusal-cervical) direction of the bracket, performing closing movement and smooth twist of the pliers.

**Figure 2:**
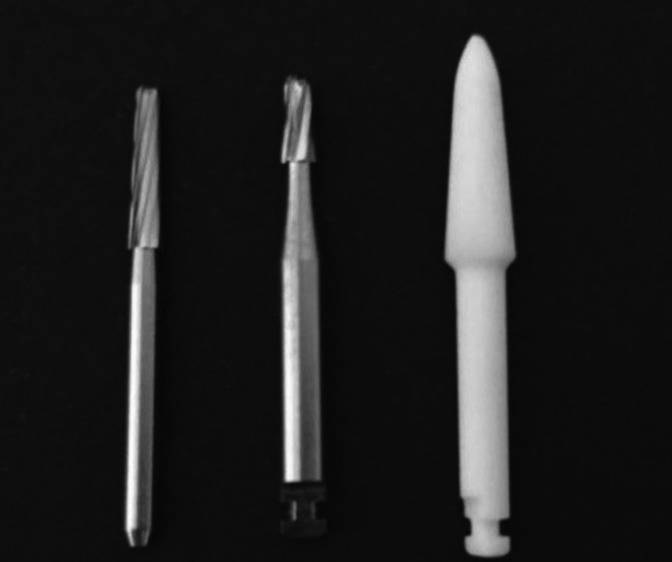
Drills for removal of remaining adhesive.

After bracket debonding, the adhesive remnants were removed from the enamel surface in group 1 by using a tungsten carbide 24-blade high-rotation drill #CF 375R (Orthometric, Marília, SP, Brasil); in group 2 by using a tungsten carbide 9-blade low-rotation drill #CB 27 (Orthometric); and in group 3 by using a Fiberglass® #2 ¬– TDV low-speed glass fiber drill (Fig. 2). In all three groups, drilling was performed without irrigation, and the drills were positioned parallel to the long axis of the tooth, making lateral movements in the mesiodistal direction of the crown, for 30 seconds in group 1, and 50 seconds in groups 2 and 3. 

After removal of the adhesive remnants from the enamel surface, grooves were made as described in the previous step, thus obtaining a cross-section of the bonding region of the brackets, allowing for microscopic visualization of the enamel prisms.

The fragments were then processed for scanning electron microscopy, obtaining electron micrographs with magnification of 150 X, 500 X, and 2,000 X. A qualitative evaluation was conducted as well.

## Results

**Figure 3:**
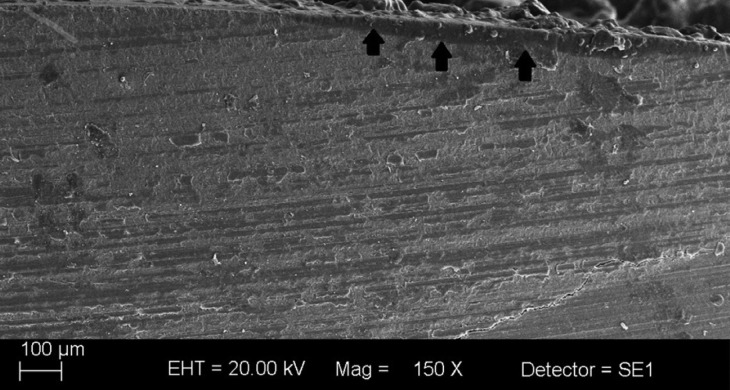
Presence of adhesive remnants on the enamel surface.

The micrographs obtained by scanning electron microscopy demonstrated that, using the proposed protocols, all tested methods were inefficient, given the presence of adhesive remnants in all groups after cleaning the enamel surface using the drills corresponding to each group (Fig. 3).

Group 1, in which a high-rotation, multi-laminated drill was used, presented the least amount of adhesive remnants on the enamel structure, followed by groups 2 and 3, respectively.

**Figure 4:**
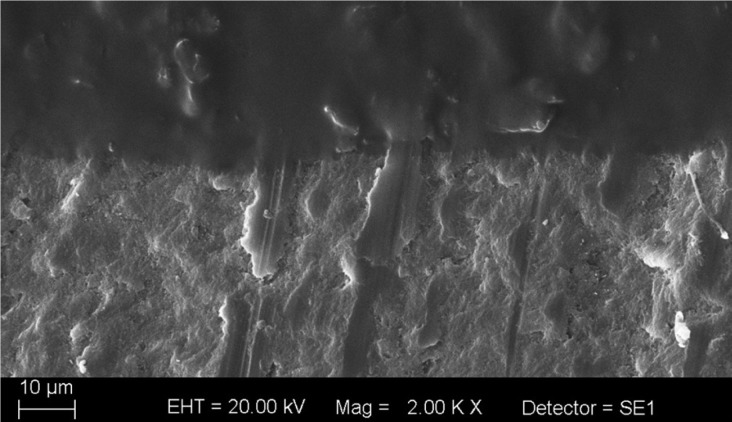
Adhesive remnants on the enamel surface in group 3.

Group 3, in which a low-rotation fiberglass drill was used, presented the highest amount of adhesive remnants on the enamel structure, not only visible during microscopy analysis (Fig. 4), but also through visual inspection, which shows clinical inapplicability.

## Discussion

Several studies have been reported in the literature regarding roughness and superficial topography evaluation of the enamel surface after bonding and debonding of orthodontic brackets (1,5,6,10-13). However, few studies (14) have been concerned with the persistence of adhesive remnants, incorporated into the enamel structure, after bracket detachment procedures and enamel surface cleaning.

 Enamel acid etching as a preparation for the bracket bonding promotes changes in the morphology of its surface, and in a mean conditioning time of thirty seconds, a typical pattern of honeycomb or hive is formed (15).

The penetration of the adhesive agent after acid etching forms tags of resinous material incorporated into the enamel (16). According to the etching protocol used, different saturation and depth patterns of these resin tags will form on the enamel. Projection depths from 9 to 28 micrometers may be observed, when acid etching is made during 15 to 60 seconds, respectively (17).

After completion of orthodontic therapy and removal of the brackets, the final goal is to remove the adhesive remnants from the enamel surface, restoring its initial pretreatment configuration. According to Cardoso *et al.* (6), the ideal material to remove the adhesive remnants from the dental enamel must have a greater hardness than that of the adhesive, and smaller than that of the enamel. However, according to Zarrinnia *et al.* (3), the removal of the adhesive remnants can cause an erosion depth of about 19 μm on the enamel surface.

It should be highlighted that resinous material may remain embedded into the enamel, even after bracket removal and enamel cleaning and polishing. Bishara *et al.* (4) have claimed that the enamel gloss achieved by removing the adhesive remnants and polishing the surface does not mean total absence of adhesive infiltrated into the enamel.

In agreement with other investigations (4,9,13), this study verified the presence of adhesive remnants incorporated into the enamel after bracket detachment and enamel surface cleaning.

According to Leão Filho *et al.* (9), many studies have been conducted to evaluate the surface topography of dental enamel after bracket debonding and enamel cleaning and polishing. However, according to those authors, few studies have studied whether the resinous infiltrate of the adhesive system remains incorporated into the enamel after the finishing and polishing procedures at the end of the orthodontic treatment.

This study found that the tungsten carbide multi-laminated, high-rotation drill presented the best results in relation to the adhesive remnant removal after the bracket debonding. These results are in agreement with those of Leão Filho *et al.* (9), who have compared only multi-laminated, high- and low-rotation drills.

Although Ryf *et al.* (13) have demonstrated that enamel surface cleaning after bracket debonding by using solely high-rotation multi-laminated drills provoke enamel structure loss, Janiszewska-Olszowska *et al.* (1) , conducting a systematic review of the literature regarding the effect of the orthodontic bracket detachment and the removal of the adhesive remnants, have demonstrated that high-rotation tungsten carbide drills are the most commonly used because they are more effective and require shorter working time as compared to other methods. The authors of that study have concluded that orthodontic treatment with bracket bonding causes irreversible damage to the dental enamel, independently of the protocol used to remove the adhesive remnants.

Different findings were achieved by Cardoso *et al.* (6) and Macieski *et al.* (11), who have evaluated positively the clinical use of low-rotation glass fiber drills to remove the adhesive remnants from the enamel surface after bracket debonding. The results can be attributed to the fact that these two studies evaluated only the quality of roughness and enamel surface polishing after bracket debonding, and not the presence of adhesive remnants incorporated into the enamel.

## Conclusions

• All methods evaluated in this study have proved to be inefficient for total removal of the adhesive remnants from the enamel.

• The tested method showed that the best effectiveness for the removal of the adhesive remnants after bracket debonding was the use of a tungsten carbide multi-laminated, high-rotation drill, followed by the use of a tungsten carbide multi-laminated, low-rotation drill.

• The use of fiberglass drill alone has proved to be inefficient for clinical use, given the large amounts of adhesive remnants it leaves on the enamel.
